# Case Report: Bilateral testicular teratoma in an infant with torsion of an intra-abdominal testis and contralateral testicular teratoma

**DOI:** 10.3389/fped.2025.1680506

**Published:** 2025-11-03

**Authors:** Chaoyang Hua, Hongjie Fan, Zhan Guo, Xing Li, Yanfang Yang, Jianpeng Bi

**Affiliations:** Department of Urology, Children’s Hospital Affiliated of Zhengzhou University, Henan Children’s Hospital, Zhengzhou Children’s Hospital, Zhengzhou, Henan, China

**Keywords:** bilateral testicular teratoma, intra-abdominal testis, testicular torsion, testis-sparing surgery, infant oncology, cryptorchidism

## Abstract

**Background:**

Bilateral testicular tumors in infants are extremely rare. This case report describes synchronous bilateral mature teratomas complicated by torsion of an intra-abdominal undescended testis (IAT), and underscores the clinical importance of early diagnosis, timely surgical intervention, and fertility-preserving management, providing valuable reference for future cases.

**Case presentation:**

A 3-month-old boy presented with an empty right hemiscrotum. Imaging revealed a right intra-abdominal mass (22.8 × 15.9 × 21.3 mm) and left testicular lesion (7.1 × 3.9 × 7.0 mm). Serum alpha-fetoprotein was within normal limits for age, suggesting benign disease. Laparoscopy confirmed a torsed necrotic right testicular mass, managed by orchiectomy. Left testis-sparing surgery excised a separate tumor. Histopathology confirmed bilateral mature teratoma. Hormonal profiles, including testosterone (0.81 ng/mL) and follicle-stimulating hormone (3.74 mIU/mL), as well as karyotype (46,XY), were normal. No additional therapy was required, as mature teratomas are benign. Postoperative alpha-fetoprotein levels normalized, with no recurrence at 6-month follow-up. Parental education regarding testicular examination is important for early detection of future abnormalities.

**Conclusions:**

Tumors associated with intra-abdominal undescended testes warrant urgent intervention due to torsion risk. Surgery preserving testicular tissue is recommended for bilateral benign teratomas to maintain fertility. Serial alpha-fetoprotein monitoring and ultrasound surveillance are essential postoperatively.

## 1 Background

Pediatric testicular tumors are uncommon, with an incidence of 0.5–2 per 100,000, accounting for approximately 1% of solid tumors in children (1). Known risk factors for pediatric testicular tumors include cryptorchidism, genetic abnormalities, and family history of germ cell tumors. Bilateral testicular tumors are exceptionally rare in pediatric patients, and only a few cases have been reported in the literature (2–13). Cryptorchidism is a common congenital condition in prepubertal children and can be classified clinically as a palpable or nonpalpable testis. The intra-abdominal testis (IAT) is a common type of nonpalpable testis, but IAT combined with testicular tumors is exceedingly rare, and only a few reports have documented testicular tumors in cases of IAT (14). In this report, we report a unique case of synchronous bilateral mature teratomas, with torsion of an intra-abdominal undescended right testis, highlighting the importance of early detection, timely surgical intervention, and fertility preservation.

## 2 Case presentation

### 2.1 History and clinical data

**Figure 1:**
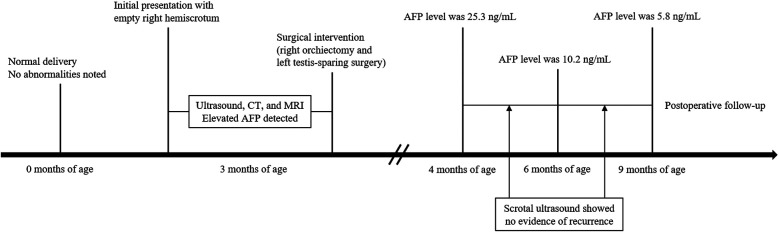
Timeline of the clinical course, diagnostic evaluations, surgical management, and postoperative follow-up in the present case.

A timeline of the patient's clinical course is summarized in Figure 1, including initial detection, imaging studies, surgical interventions, and follow-up assessments. A 3-month-old boy was brought to medical attention due to a persistently empty right hemiscrotum noted since birth, without acute symptoms such as irritability or pain. Physical examination revealed a poorly developed and empty right hemiscrotum, with no palpable testis in the right inguinal region. The left testis was palpable within the scrotum, enlarged, and contained a discrete mass.

### 2.2 Imaging studies

**Figure 2:**
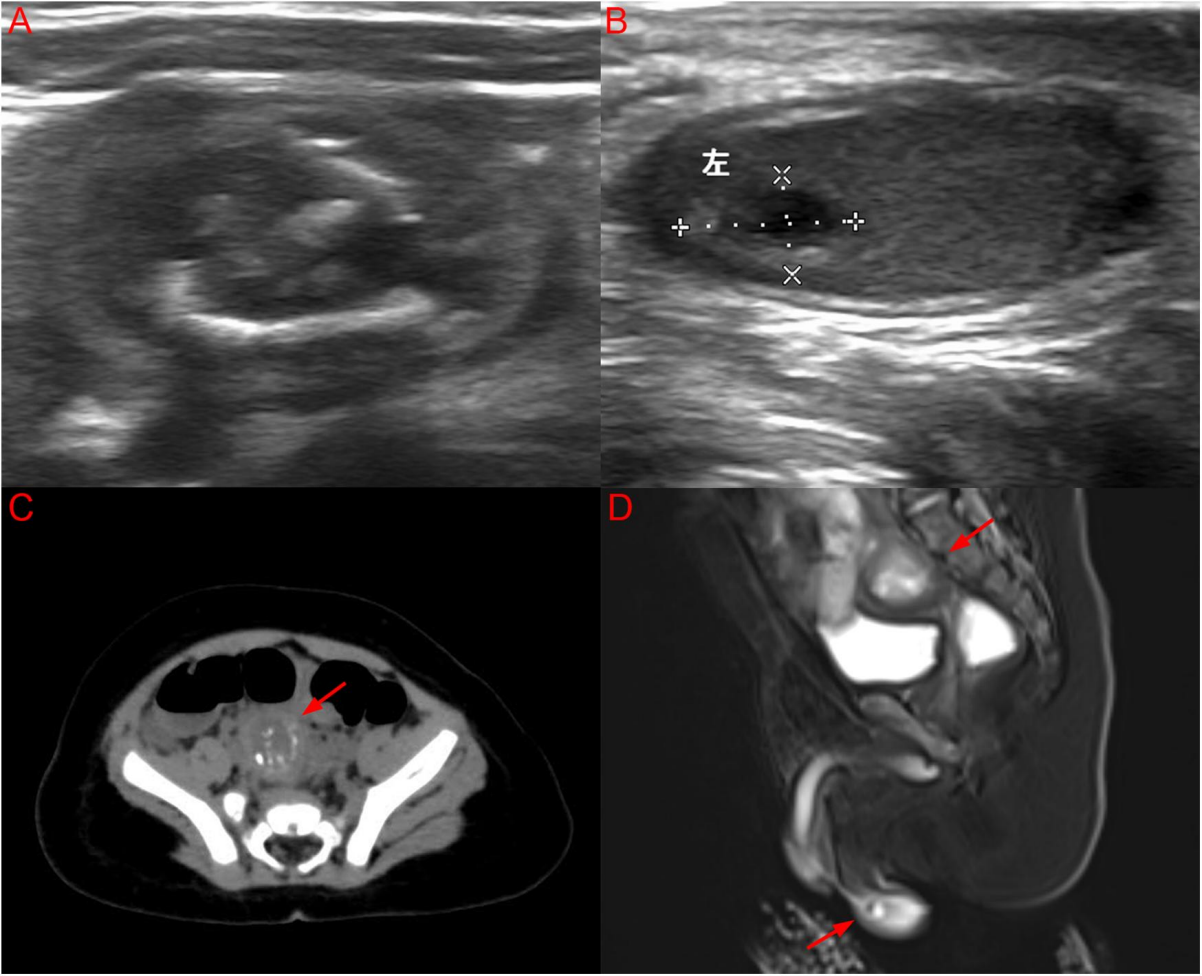
**(A)** US image of a well-defined mixed echogenic mass in the lower right abdomen; **(B)** US image of a cystic solid mass lesion in the left testis; **(C)** CT image of a mixed density mass located above the bladder with an arrow; **(D)** MRI image of a right IAT with a mixed mass and a mass in the left testis synchronously with arrows. (Color version available online.).

An ultrasound (US) scan revealed a heterogeneous 22.8 × 15.9 × 21.3 mm mass in the lower right abdomen (Figure 2A) and an additional 7.1 × 3.9 × 7.0 mm heterogeneous, predominantly cystic lesion in the left testis (Figure 2B). Computed tomography (CT) revealed a mixed-density mass located above the bladder (Figure 2C). The lesion demonstrated both cystic and solid components, with areas of calcification, but no evidence of invasion or distant metastasis on thoracoabdominal CT. MRI was performed to better delineate tissue composition and vascularity, complementing CT for surgical planning. Magnetic resonance imaging (MRI) revealed a right IAT with a mixed mass and a mass in the left testis (Figure 2D).

### 2.3 Laboratory results

Laboratory tests showed an elevated alpha-fetoprotein (AFP) level (84.28 ng/mL; physiological range in infants <1 year: typically <100 ng/mL). AFP was therefore interpreted as within the physiological range for age, favoring a benign lesion rather than malignancy. Additional hormonal profiles were within normal limits for age: follicle-stimulating hormone 3.740 mIU/mL, luteinizing hormone 4.100 mIU/mL, prolactin 19.100 ng/mL, estradiol <5.000 pg/mL, testosterone 0.810 ng/mL, and progesterone 0.653 ng/mL. Serum human chorionic gonadotropin (hCG) was <0.100 mIU/mL. Chromosomal analysis revealed a 46,XY karyotype with positive SRY gene. Preoperative differential diagnosis included teratoma, yolk sac tumor, and other germ cell tumors. This assessment relied on serum tumor markers, imaging (ultrasound and MRI), and the patient's clinical context. Notably, AFP was within the normal range for infants under 1 year, and imaging revealed heterogeneous, predominantly cystic lesions, favoring a benign teratoma over yolk sac tumor or malignant germ cell tumor.

### 2.4 Surgical procedure and outcome

**Figure 3:**
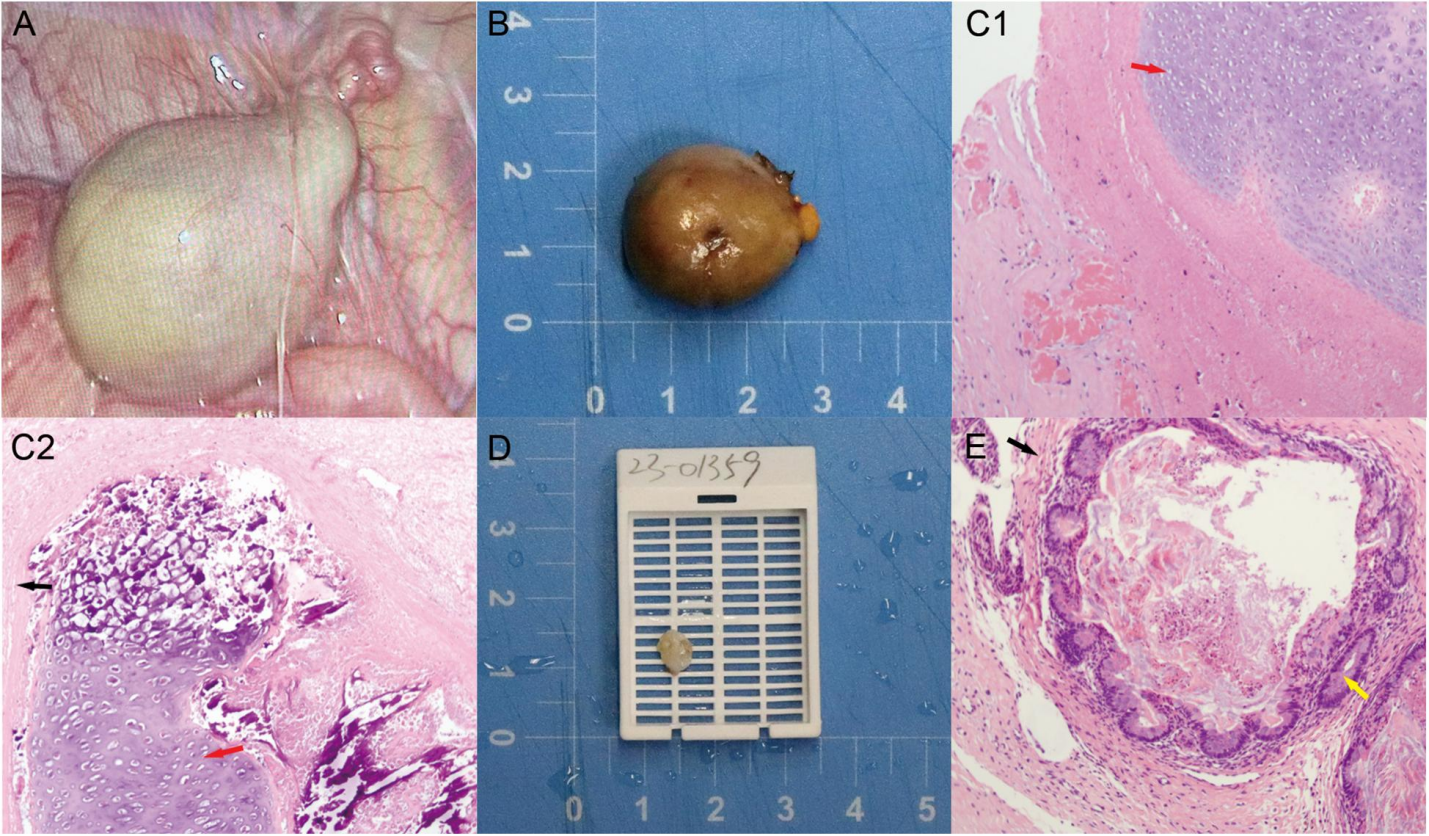
**(A)** Laparoscopic exploration: right IAT mass with torsion on the right internal ring; **(B)** grayish-white, round solid mass lesion, right testis (scale bar = 1 cm); (C1–2) extensive necrotic chondrocytes with focal calcification in the right testicular mass (red arrow: mesodermal tissue—immature cartilage; black arrow: ectodermal tissue—epithelium); **(D)** cystic solid mass in the left testis (scale bar = 1 cm); **(E)** enterocytes in the left testicular mass (yellow arrow: endodermal tissue—intestinal gland). Color version available online.

Laparoscopic exploration of the abdomen revealed a 25 × 23 × 24 mm grayish-white, round mass located on the inner side of the right internal ring above the posterior bladder (Figure 3A). The right testis was found to have undergone complete torsion of the vascular pedicle, with congested and necrotic spermatic cord structures. The degree of torsion could not be precisely measured, but the ischemic changes indicated a prolonged duration. The contralateral deep inguinal ring was inspected and appeared normal. No testis was found in the lower pole of the right kidney. The right testis was confirmed to have undergone torsion and necrosis, possibly associated with the testicular mass, and orchiectomy was performed. Orchiectomy was chosen over detorsion because the right testis was entirely necrotic and non-salvageable.

The excised tissues were sent for rapid histopathological analysis (Figure 3B), and the results indicated torsion and extensive necrosis of the pedunculated mature teratoma on the right side, with focal calcification (Figure 3C1–2).

Next, after the left scrotum and testicular tunica albuginea were incised, the testicular tumor was found to be located in the upper pole of the testicular parenchyma, with clear boundaries. The tumor was approached through a scrotal incision. The majority of the normal testicular parenchyma was preserved, and careful dissection ensured preservation of testicular vascularity. Intraoperative frozen section analysis was performed to confirm tumor-free margins. Testis-sparing surgery (TSS) was also performed.

Finally, orchidopexy was performed to secure the left testis within the scrotum. The excised tumor tissue was also sent for rapid histopathological analysis (Figure 3D). The results also revealed a mature teratoma on the left side (Figure 3E). The final pathology of the tumoral specimen was consistent with the intraoperative rapid frozen section results. Postoperative management involved routine wound care without adjuvant chemotherapy or radiotherapy, as the histopathology confirmed benign mature teratoma.

**Figure 4:**
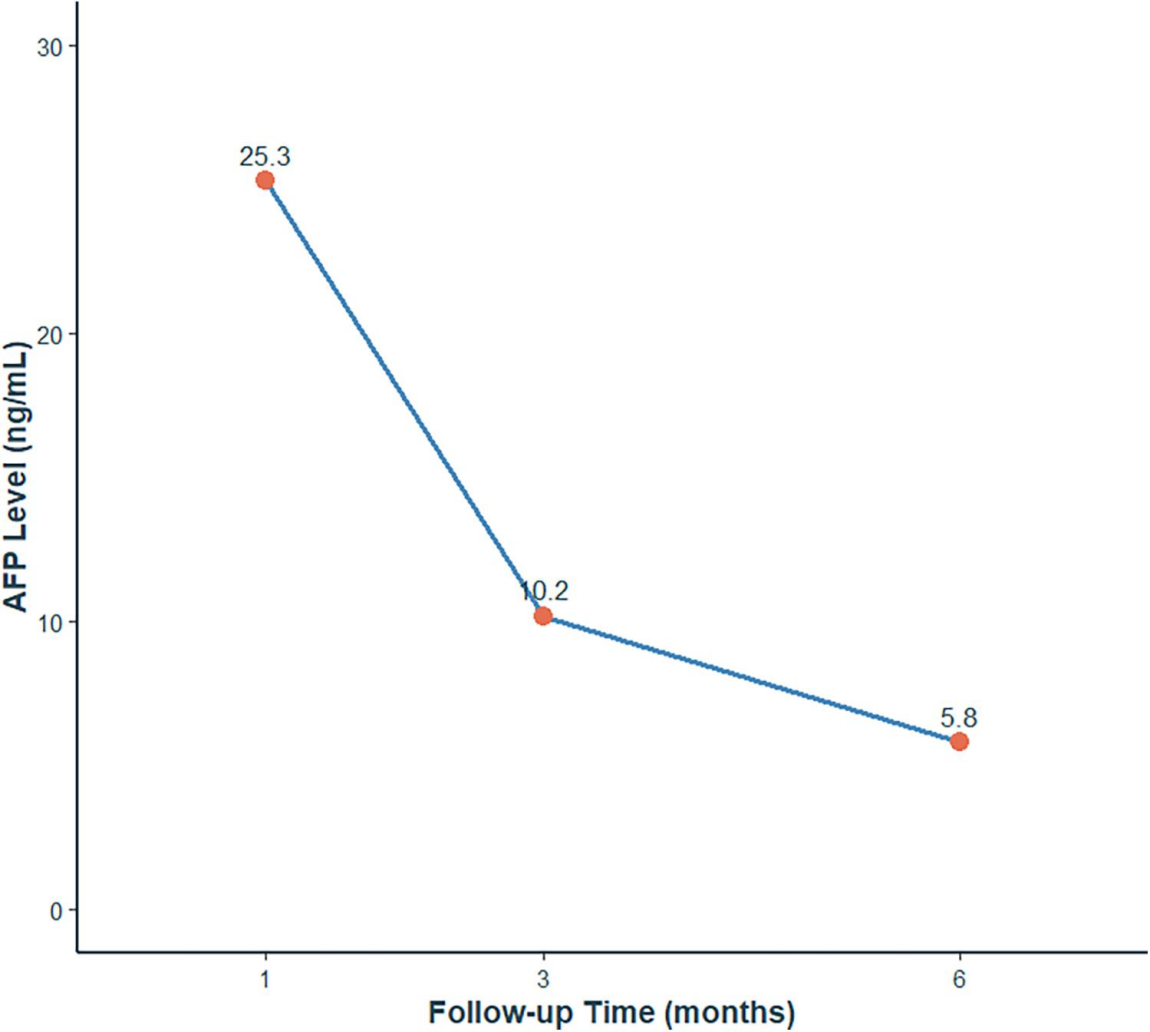
Postoperative AFP level changes at 1, 3, and 6 months.

**Figure 5:**
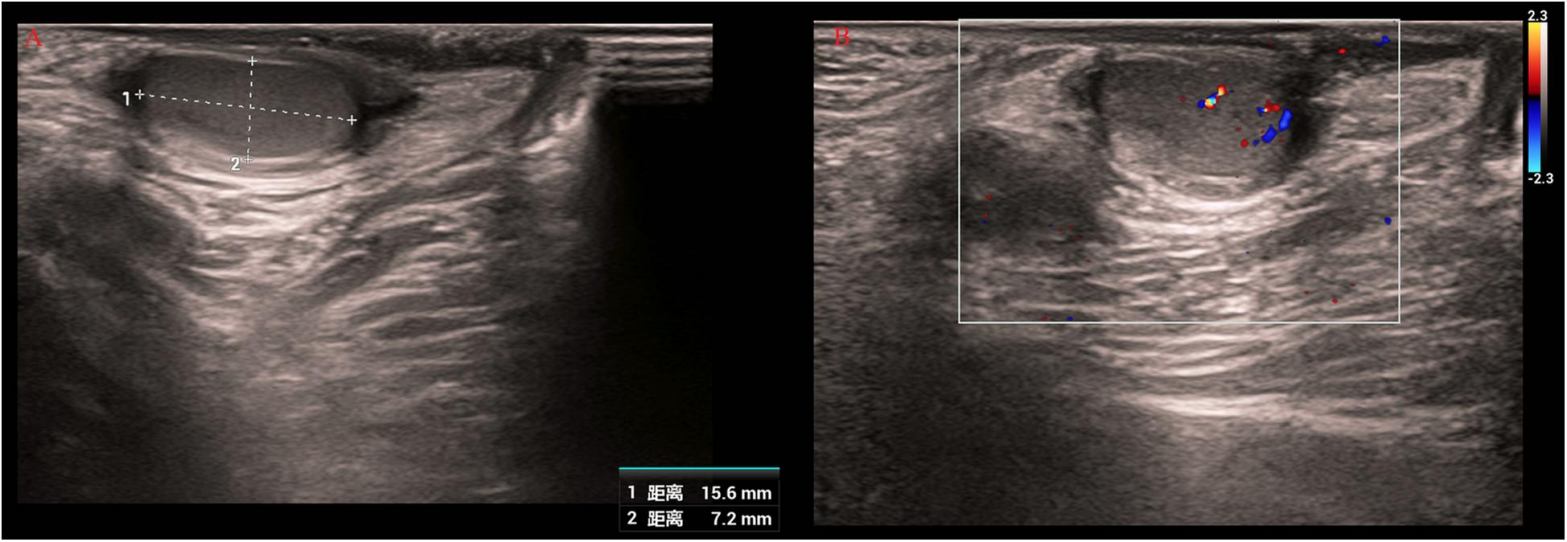
Postoperative scrotal ultrasound of the left testis. **(A)** Gray-scale image showing preserved parenchyma with normal echogenicity. **(B)** Color Doppler image demonstrating normal vascular flow without evidence of residual tumor or recurrence.

Serial AFP measurements demonstrated normalization of levels (postoperative values: 25.3 ng/mL at 1 month, 10.2 ng/mL at 3 months, 5.8 ng/mL at 6 months), consistent with both surgical resolution and age-related physiological decline (Figure 4). Postoperative scrotal ultrasound of the left testis demonstrated preserved parenchyma with normal echogenicity (Figure 5A) and normal vascular flow on color Doppler imaging (Figure 5B), with no evidence of residual tumor or recurrence. Follow-up at 1 month, 3 months, and 6 months after surgery, along with testicular ultrasound, revealed no evidence of recurrence.

Immunohistochemistry was deemed unnecessary, as the classic morphology confirmed a benign mature teratoma without atypical or malignant features. The lesion contained ectodermal elements (stratified squamous epithelium and neural tissue), mesodermal elements (cartilage, adipose tissue, and smooth muscle), and endodermal elements (glandular structures resembling respiratory and intestinal epithelium), with focal calcification. Representative micrographs are shown in Figure 3C1–2,E.

### 2.5 Literature review

We conducted a literature review by searching databases to identify relevant cases of bilateral testicular tumors in children. Finally, a total of 12 relevant articles were identified, comprising 12 cases of pediatric bilateral testicular tumors (Table 1).

**Table 1:** Published bilateral testicular tumor cases.

Authors, date	Age (m or y)	Cryptorchidism	Preoperative examination	AFP ng/mL	Tumor size (cm)	Operation incision	Pathology result	Treatment	Follow-up (m)	Recurrence
Royal et al. (1994) (2)	2y	No	CT	–	L: –	L: II	L:MT	L: orchiectomy	–	–
					R: –	R: II	R: YST(95%), MT(5%)	R: orchiectomy		
Mansfield et al. (1995) (3)	15m	No	US	Normal	L:1.5	L: II	L: MT	L: orchiectomy	12	No
					R:0.4	R: SI	R: EC	R: TSS		
Luo et al. (1998) (4)	7m	No	US	>10,000	L:10	L: II	L: YST	L: orchiectomy	3	No
					R:4	R: II	R:MT	R: orchiectomy		
Tasaki et al. (1998) (5)	1y	No	US	Normal	L:2.5	–	L: MT	L: orchiectomy	16	No
					R:0.8	–	R: MT	R: TSS		
Yokomizo et al. (2001) (6)	19m	No	US	646	L: –	L: II	L: YST	L: orchiectomy	9	No
					R:2.0	R: II	R: YST	R: orchiectomy		
Herek et al. (2004) (7)	10m	No	US	37.6	L:1.9	L: II	L: MT	L: orchiectomy	36	No
					R:0.6	R: II	R: MT	R: TSS		
Koski et al. (2008) (8)	20m	No	US	Normal	L:2.6	L: II	L: MT	L: TSS	6	No
					R:1.4	R: II	R: MT	R: TSS		
David et al. (2011) (9)	37w	L: Yes	US, MRI	Normal	L:10.5	L: LP	L: JGCT	L: orchiectomy	17	No
		R: Yes			R:9.5	R: LP	R: JGCT	R: orchiectomy		
Madden et al. (2012) (10)	11y	No	US	Normal	L:1.3; 1.0	L: II	L: DC, EC	L: TSS	6	No
					R:0.5	R: II	R: EC	R: TSS		
Dong et al. (2018) (11)	7m	No	US	243	L:1.6	L: II	L: YST	L: orchiectomy	28	No
					R:1.2	R: SI	R:MT	R: TSS		
Li et al. (2019) (12)	19m	No	US, MRI	2,122.3	L: -	L: -	L: YST	L: orchiectomy	84	No
					R:0.6	R: II	R: MT	R: TSS		
Kebudi et al. (2020) (13)	23m	No	US	4,356	L:0.6	L: II	L: MT	L: TSS	1.5	No
					R:2.5	R: II	R: YST	R: orchiectomy		
Present case, 2023	3m	L: No	US, CT, MRI	84.28	L:0.7	L: SI	L: MT	L: TSS	3	No
		R: Yes			R:2.2	R: LO	R: MT	R: orchiectomy		

YST, yolk sac tumor; MT, mature teratoma; JGCT, juvenile granulosa cell tumors; TS, testicular seminoma; EC, epidermoid cyst; DC, dermoid cysts; TSS, testis sparing surgery; II, inguinal incision; SI, scrotal incision; LP, laparotomy; LO, laparoscope; –, not report.

## 3 Discussion

Testicular tumors account for approximately 1%–2% of solid tumors in children and typically present as unilateral cases (1). Thus, the occurrence of bilateral testicular teratomas, with the presence of an IAT-associated teratoma on one side in the current case report, is relatively rare. Since the first reported case by Rosenblum in 1948, there have been several publications on testicular tumors, and overall, bilateral testicular germ cell tumors (TGCTs) account for 1%–3% of all cases (15). Ghazarian et al. reported that the survival rate of children with TGCTs can exceed 95%; however, in recent years, the incidence of testicular tumors has increased (16), potentially due to improved diagnostic imaging, heightened awareness, environmental exposures, or genetic predispositions, and delayed diagnosis and treatment have resulted in tumor progression in some cases (16).

Cryptorchidism, particularly intra-abdominal testes (IAT), has a well-documented association with pediatric testicular teratomas, rather than germ cell tumors in general. Yam et al. suggested that cryptorchidism, especially intra-abdominal testis (IAT), is closely related to the occurrence of testicular tumors and that the majority of IAT-associated testicular tumors in prepubertal children are mature teratomas (17). Doi et al. proposed that IAT tumors located at the internal inguinal ring may hinder the normal descent of the testis, indicating a specific relationship between IAT and teratoma development (18). Nguyen et al. reported that approximately 64% of patients with torsion of the IAT had associated testicular tumors (19). Among the 12 previously reported bilateral pediatric cases, only one involved cryptorchidism, whereas our case demonstrates synchronous bilateral mature teratomas with right IAT and torsion, emphasizing the clinical importance of early detection and monitoring in patients with undescended testes. Therefore, early detection and timely treatment are crucial for pediatric IAT tumors.

In recent years, as more studies have been published, there have been differing viewpoints on the relationship between cryptorchidism and testicular tumors. Leslie et al. mentioned in their recent publication that cryptorchidism could increase the risk of TGCTs, but the overall risk was less than 1% (20). Among the 12 cases reviewed in the literature, only one case reported cryptorchidism combined with a testicular tumor. However, we report a case involving bilateral testicular tumors with concurrent right IAT with torsion and necrosis. To the best of our knowledge, this is the first reported case of its kind. Therefore, further research is needed to confirm whether cryptorchidism, especially IATs, is indeed associated with the occurrence of testicular tumors.

More than 90% of pediatric testicular tumors present as painless scrotal masses, making early detection challenging (21). For the diagnosis of pediatric testicular tumors, in addition to routine physical examinations, US is the preferred auxiliary diagnostic tool, with a sensitivity of up to 100% (21). However, it cannot fully distinguish between benign and malignant tumors. Serum AFP plays a significant role in discriminating between benign and malignant tumors. Differential diagnosis between testicular teratoma and other germ cell tumors, such as yolk sac tumor, relies on serum tumor markers, imaging, and histopathology (22). In prepubertal patients, teratomas are generally benign, whereas yolk sac tumors typically show markedly elevated AFP and distinct histological features. In our case, normal AFP and characteristic histopathology confirmed bilateral mature teratomas. Notably, AFP levels may be physiologically elevated in children under one year of age and typically remain below 100 ng/mL, but an elevated AFP level suggests a greater possibility of malignancy in children (13). Therefore, preoperative assessment, including scrotal US, AFP level, and CT evaluations, is crucial in the diagnosis of pediatric testicular tumors, as the results play a significant role in guiding the choice of surgical procedure.

For pediatric testicular tumors, the current preferred first-line treatment is surgical excision, but there are different surgical approaches. Preoperative ultrasound revealed heterogeneous testicular masses with cystic and solid components and significantly elevated AFP levels, suggesting inguinal radical orchiectomy. For prepubertal children with suspected benign testicular tumors, testis-sparing surgery (TSS) is recommended whenever feasible. If postoperative histopathology confirms a mature teratoma, no further adjuvant treatment is necessary, and imaging follow-up can be individualized. Kebudi et al. recommended bilateral radical orchiectomy when the preoperative diagnosis indicated malignancy in both testes. If the diagnosis suggests a benign tumor, only unilateral or bilateral TSS is recommended (13). Owing to the implications for future fertility in pediatric patients, the preoperative diagnosis and surgical treatment plan for bilateral testicular tumors should be approached with extreme caution. Previous reports of bilateral testicular tumors in infants have demonstrated several common features: most cases occurred in early childhood, with mature teratoma being the predominant benign histology. Serum AFP levels were variably elevated, depending on the presence of malignant components, but often remained within the physiological range in benign cases. Testis-sparing surgery was attempted whenever benign pathology was suspected, whereas orchiectomy was chosen when the testis was non-viable or malignancy was strongly suspected (23). Differences among the published cases mainly related to tumor location (scrotal vs. intra-abdominal), association with cryptorchidism, and the extent of surgical intervention. Compared with these reports, our case is unique in presenting with synchronous bilateral mature teratomas and torsion of an intra-abdominal testis, underscoring the importance of early recognition, prompt surgery, and fertility-preserving strategies when feasible.

This case highlights several important learning points for clinicians: early detection of intra-abdominal testis (IAT) is essential to prevent torsion and necrosis; preoperative assessment combining physical examination, imaging, and serum tumor markers is critical for differentiating benign from malignant tumors; intraoperative frozen section played a key role in guiding the surgical decision, particularly in determining the feasibility of testis-sparing surgery in the context of bilateral disease; testis-sparing surgery should be prioritized for benign lesions to preserve fertility; and serial postoperative surveillance with AFP and ultrasound is important for monitoring recurrence and testicular function. Postoperative surveillance should include serial AFP measurements and scrotal ultrasound at regular intervals (1, 3, 6 months postoperatively), with assessment of testicular volume and vascularity to monitor function. Additionally, reporting this unique case of synchronous bilateral mature teratomas with torsion of an intra-abdominal testis provides valuable reference for the management of similar rare pediatric cases. In addition, parental education regarding testicular examination is essential to facilitate early detection of future abnormalities.

We acknowledge that this follow-up period is relatively short. Long-term surveillance is planned, including annual scrotal ultrasound and AFP measurement until puberty, to monitor for tumor recurrence and assess testicular function. Fertility follow-up may be considered in adolescence to ensure normal endocrine and reproductive capacity. Another limitation is that immunohistochemistry was not performed. Although the classic morphology confirmed a benign mature teratoma without atypical or malignant features, immunohistochemistry could help exclude rare malignant elements in ambiguous cases, and its absence should be acknowledged. The occurrence of bilateral tumors at such a young age naturally raises the question of underlying genetic predisposition. Although no genetic testing was performed in this case, future studies should investigate potential hereditary or molecular factors contributing to pediatric bilateral testicular tumors.

## 4 Conclusion

Pediatric bilateral testicular tumors are exceptionally rare, particularly when coexisting with IAT tumors. Early intervention is critical to prevent torsion and necrosis. Ultrasound remains the preferred diagnostic tool. Surgical excision is primary, with TSS favored for bilateral teratomas to preserve fertility. Rigorous postoperative surveillance is essential, especially given fertility implications.

## Data Availability

The original contributions presented in the study are included in the article/Supplementary Material, further inquiries can be directed to the corresponding authors.
